# The effect of an exercise intervention on aerobic fitness, strength and quality of life in children with haemophilia (ACTRN012605000224628)

**DOI:** 10.1186/1471-2326-6-2

**Published:** 2006-05-29

**Authors:** Carolyn R Broderick, Robert D Herbert, Jane Latimer, Julie A Curtin, Hiran C Selvadurai

**Affiliations:** 1 School of Medical Sciences, Faculty of Medicine, University of New South Wales, Sydney 2052, Australia; 2 Children's Hospital Institute of Sports Medicine, The Children's Hospital at Westmead, Westmead, NSW 2145, Australia; 3 School of Physiotherapy, University of Sydney, Lidcombe NSW 1825, Australia; 4 Department of Haematology, The Children's Hospital at Westmead, Westmead, NSW 2145, Australia; 5 Department of Respiratory Medicine, The Children's Hospital at Westmead, Westmead, NSW 2145, Australia

## Abstract

**Background:**

Children with haemophilia have lower levels of fitness and strength than their healthy peers. We present the protocol of a study designed to determine whether an exercise intervention improves quality of life, aerobic fitness and strength in children with haemophilia.

**Methods/Design:**

The study will be a randomised, assessor-blinded, controlled trial of exercise treatment. Seventy children aged between 6 and 18 years with haemophilia or von Willebrand disease will be recruited from two paediatric haemophilia clinics in NSW. Each participant will be allocated to an exercise group or a control group using a concealed allocation procedure. The control group will receive usual medical care while the intervention group will receive usual medical care plus an exercise program for 12 weeks. Outcomes (VO_2peak_, knee extensor strength and quality of life) will be measured at baseline and on completion of the exercise program by a blinded assessor. The primary analysis will be conducted on an intention to treat basis. The effects of the exercise intervention on each of the three primary outcomes will be estimated from between-group differences in the mean outcome adjusted for baseline scores.

**Discussion:**

This study will be the first randomised controlled trial to examine the effects of a structured exercise program on fitness and quality of life in children with haemophilia.

## Background

Haemophilia A and B are X-linked recessive conditions which affect 1 in 7,000 males in Australia [1]. They are characterised by deficiency of Factor VIII or Factor IX (in the mild and moderate forms) and absence of Factor VIII or IX (in the severe form). Haemophilia is characterised by an increased bleeding tendency, the severity of which is dependent on the absolute levels of Factor VIII or IX. Mild haemophilia (when the factor level is 5–40%) is rarely associated with spontaneous bleeds. Moderate haemophilia means that the factor level is <5% of normal and severe haemophilia occurs when the factor level is <1%. The hallmarks of haemophilia are intramuscular and intra-articular bleeds. Some children develop "target joints" characterised by multiple bleeds into the same joint which can lead to destruction of the joint surfaces (haemophilic arthropathy).

In the past, children with haemophilia were often discouraged from physical activity due to a concern that this would increase the risk of haemarthroses. The availability of recombinant factor XIII has meant that children with severe haemophilia can now have prophylactic therapy to prevent spontaneous bleeds. A multi-centre study comparing prophylactic therapy with on-demand treatment in young adults with severe haemophilia demonstrated fewer joint bleeds and lower levels of arthropathy in the prophylaxis group [2].

Even though children with haemophilia are now advised to participate in physical activity, a number of studies, including recent studies, have suggested that these children have lower levels of fitness and strength when compared with their healthy peers [3,4]. Unfortunately, these studies have used samples that are not representative (they sample small numbers of volunteers from haemophilia clinics) and it therefore remains unclear whether children with haemophilia do in fact have lower levels of fitness.

The therapeutic role of exercise in children with haemophilia has not been well documented but there is some evidence that exercise can improve coagulation parameters. Factor VIII levels have been shown to increase after a single strenuous bout of exercise in non-haemophiliacs [5,6]. A modest improvement in coagulation parameters after an acute bout of exercise has also been shown in children with mild and moderate haemophilia [7]. Whether this improvement in coagulation parameters correlates with frequency and severity of bleeding episodes remains unclear. One pilot study which examined the effect of resistance training on 5 adults with haemophilia reported this type of training improved strength and reduced the frequency and severity of bleeding episodes [8]. However, the findings of this small and uncontrolled study cannot be considered reliable. The effects of resistance training on frequency and severity of bleeds needs to be tested in a larger randomised controlled trial.

Studies which report positive effects from an exercise intervention in adults with haemophilia have had very small subject numbers and lacked experimental controls [8]. Hilberg et al. [9] documented improvements in isometric leg strength and proprioception in *adults *with severe haemophilia following a physical training programme, but this study had small numbers and was not randomised and therefore gives limited information about the effect of exercise on muscle performance.

Finally, the effect of an exercise programme on quality of life in children with haemophilia has yet to be examined. In other chronic diseases in children, however, exercise interventions have shown positive effects on quality of life. Selvadurai et al. [10] reported improvements in quality of life, as measured by the Quality of Well Being Scale, in children with cystic fibrosis following an in-hospital aerobic exercise programme. Recently a disease-specific quality of life questionnaire (Haemo-QoL) has been validated in children with haemophilia [11-13]. The effect of an exercise programme on quality of life can now be assessed.

The primary aim of this study is to determine the effects of exercise on aerobic fitness, strength and quality of life in children with haemophilia. A secondary aim is to examine the effect of an exercise programme on the frequency and severity of bleeding episodes.

## Study design

This study will be a randomised, assessor-blinded, controlled trial of exercise treatment for children with haemophilia. The control group will receive usual medical care while the intervention group will receive a general exercise program of 12 weeks duration. Outcomes will be measured at baseline, on completion of the exercise program at 3 months and at 6 months.

Seventy children with Haemophilia A or B or von Willebrand disease who are currently managed through the haematology clinic at The Children's Hospital at Westmead and Sydney Children's Hospital will be recruited. To participate in the study children must be aged between 6 and 18 years, have a diagnosis of mild, moderate or severe haemophilia (A or B) or von Willebrand disease and the parent/guardian and child must agree to accept randomisation to either intervention or control group. Subjects will be excluded if they have any contraindication to aerobic or resistance training. Children who are already involved in regular, organised physical activity lasting greater than 1 hour for more than 3 sessions per week will also be excluded.

## Methods

Following the screening consultation, baseline measures will be collected by the researcher. All children will have baseline anthropometric data recorded (height, weight, BMI, and body fat using bioelectrical impedance). They will then undergo fitness testing. This will consist of tests of grip strength and isokinetic strength testing to assess quadriceps and hamstrings strength in the non-dominant leg. In addition a VO_2peak _test will be performed using a treadmill protocol. All subjects or their parents/guardians will complete the Haemo-QoL questionnaire [13] and the Habitual Activity Estimation Scale (HAES) [14]. The HAES questionnaire will be used to explain effects of exercise, should they be apparent, and to determine whether completing a structured 12 week exercise program increases levels of habitual activity.

A randomisation schedule will be created with computer-generated random numbers by a person not otherwise involved in the recruitment process. The allocation will be in randomly permuted blocks of 4, 6 and 8. Allocations will be placed in sequentially numbered, sealed, opaque envelopes to ensure concealment from the researchers recruiting subjects, and from the subjects themselves, prior to randomisation. Potential subjects (or the guardians of subjects) will be invited to participate in the study and the methods of the study will be explained to them verbally and in writing. If they agree to participate baseline measures will be obtained before the subject is randomised to a group. Subjects will be considered to have entered the trial at the time the envelope containing their allocation is opened.

Participants in the **control group **will receive usual medical care by their treating haematologist and associated health practitioners. Their management will include factor therapy (prophylaxis or "on demand"), as determined by their treating haematologist. All parents will have been educated about the disease and been taught how to recognise a bleeding episode. For the purposes of this study when defining a bleeding episode we have accepted the definition widely used by other researchers that is, a bleed is "an episode of bleeding requiring treatment with clotting factor"[15]. Parents will be encouraged to treat bleeding episodes as soon as recognized and advice about duration of therapy will be given by their treating haematologist.

Participants in the **exercise group **will receive a supervised, general exercise program in addition to their usual medical care. The exercise program will be conducted for 1 hour, two times per week for 12 weeks. The program will consist of a supervised "circuit training" programme including warm-up and warm-down and an age-appropriate mix of resistance exercises and aerobic stations. Aerobic training will consist of 30 minutes of continuous exercise at 60–70% HR_max_, depending on baseline fitness of the participant, as measured by VO_2peak _test. Resistance training will follow the traditional "set system" and involve 20 minutes of upper and lower limb exercises using isotonic pin-loaded equipment. The training load will be set at the 8–12 repetition maximum (RM). Subjects will perform 3 sets, each to failure, with the 8–12 RM load. Throughout the training period the load will be incremented so that failure occurs at between 8 and 12 repetitions. Ten minutes per session will involve warm-up and warm-down exercises[16]. The exercise sessions will be changed frequently to avoid boredom. Patients receiving prophylactic Factor VIII will administer this prior to the exercise intervention. A qualified paediatric physical therapist will conduct and supervise the exercise program. The therapist will undergo additional training in the aetiology and management of haemophilia and understand the early management of a bleeding episode.

Participants in the control group will be advised not to change their physical activity levels during the course of the trial. In cases where this is unavoidable, a record of the changes will be kept. Several mechanisms will be used to ensure that the trial protocol is consistently applied. Protocol manuals will be developed and the researchers (haematologists, physical therapists, exercise physiologists, other outcome assessors and the trial manager) will be trained to ensure that screening, outcome assessment, allocation and treatment protocols are conducted according to the protocol. An independent researcher will randomly audit assessment and treatment sessions to determine adherence to the protocol. Retraining will be offered to treatment providers and outcome assessors not complying with the protocol.

### Outcomes

The primary outcomes will be quality of life, strength and aerobic fitness. The details are as follows:

1. **Quality of Life **using the Haemo-QoL questionnaire. This questionnaire has been validated in children with haemophilia [13]

2. **Quadriceps strength **using a Cybex isokinetic dynamometer [17]

3. **VO_2peak _**using the Bruce treadmill protocol [18]

The *secondary *outcomes are as follows:

1. **Incidence of haemarthroses and intramuscular bleeds **– self-reported for 16 weeks (12 week intervention period and 4 weeks following)

2. **Lean tissue mass, body fat **measured using bioelectrical impedance on a body composition analyser

3. **Habitual activity **using HAES questionnaire [14]

4. **Other strength measures**

- Isokinetic hamstrings strength using a Cybex isokinetic dynamometer

- Grip strength using a hand dynamometer [19]

5. **Adverse events **(other than haemarthroses) associated with exercise or physical activity. These will be elicited by open-ended questioning.

All measurements will be taken immediately after the 12 week intervention period, by an assessor blind to group allocation. (The exception is measurement of bleeding episodes, which will be monitored as described above.) Habitual activity and quality of life will be re-measured in a telephone interview at six months. The number and type of any adverse effects that occur for the duration of the study will also be recorded using open-ended questioning.

In order to improve compliance and reduce loss to follow-up, participants in the intervention and control groups will be telephoned at the beginning of each week. Participants in the both groups will be asked about bleeding episodes in the previous week. In addition, participants in the intervention group will be reminded of their exercise training schedule. All participants will be asked to keep a diary to record all bleeding episodes during and for one month following the intervention period. The final data on number of bleeds will be a composite of the data obtained from diaries and from telephone follow-ups.

### Sample size and power analysis

A sample size of 70 subjects (35 subjects per group) will provide an 80% probability of detecting an effect of 0.75 standard deviations on H-QOL scale assuming α of 0.05 and worst-case loss to follow up of 15%.

### Data analysis

Data will be analysed by a statistician who is blinded to group. All analyses will be by intention to treat. That is, outcome data will be collected regardless of compliance with the trial protocol, and each patient's data will be analysed in the group to which the subject was allocated. The effect of exercise will be estimated with analysis of covariance using a regression approach. To maximise the precision of estimates, pre-test scores of outcome variables will be entered into the regression models as covariates. The uncertainty associated with estimates of the size of effects of exercise will be quantified with 95% confidence intervals. The integrity of the trial data will be monitored by regular scrutiny of data sheets for omissions and errors. Data will be double entered and the source of any inconsistencies will be explored and resolved.

## Discussion

We have presented the rationale and design for an RCT examining the effects of exercise on quality of life, fitness and strength in children with haemophilia. This study has been designed to include key methodological features recognised to minimise bias in clinical trials. These features include: true randomisation using concealed allocation, blind outcome assessment, blind analysis of results and intention-to-treat analyses. We have also included practical measures to minimise the number of dropouts and maximise compliance with the trial protocol. The results of this trial will be available in 2008 and will provide important information likely to affect the lives of children with haemophilia.

## Abbreviations

BMI – body mass index

HAES – Habitual Activity Estimation Scale

HR max – maximum heart rate

Haemo-QoL – Haemophilia quality of life questionnaire

RM – repetition maximum

VO2 peak – peak oxygen consumption

## Competing interests

The author(s) declare that they have no competing interests.

## Authors' contributions

CB conceived the study and was involved in study design and preparation of the manuscript. RH was involved in study design. JL contributed to study design, design of the intervention and preparation of the manuscript. HS was involved in design of the clinical intervention and JC provided advice regarding care of children with haemophilia.

All authors read and approved the final manuscript.

## Pre-publication history

The pre-publication history for this paper can be accessed here:


